# Membrane Curvature Sensing by Amphipathic Helices Is Modulated by the Surrounding Protein Backbone

**DOI:** 10.1371/journal.pone.0137965

**Published:** 2015-09-14

**Authors:** Christine M. Doucet, Nina Esmery, Maud de Saint-Jean, Bruno Antonny

**Affiliations:** IPMC, CNRS UMR 7275, 660 route de Valbonne, 06560 –Valbonne, France; Institut Curie, FRANCE

## Abstract

Membrane curvature is involved in numerous biological pathways like vesicle trafficking, endocytosis or nuclear pore complex assembly. In addition to its topological role, membrane curvature is sensed by specific proteins, enabling the coordination of biological processes in space and time. Amongst membrane curvature sensors are the ALPS (Amphipathic Lipid Packing Sensors). ALPS motifs are short peptides with peculiar amphipathic properties. They are found in proteins targeted to distinct curved membranes, mostly in the early secretory pathway. For instance, the ALPS motif of the golgin GMAP210 binds trafficking vesicles, while the ALPS motif of Nup133 targets nuclear pores. It is not clear if, besides curvature sensitivity, ALPS motifs also provide target specificity, or if other domains in the surrounding protein backbone are involved. To elucidate this aspect, we studied the subcellular localization of ALPS motifs outside their natural protein context. The ALPS motifs of GMAP210 or Nup133 were grafted on artificial fluorescent probes. Importantly, ALPS motifs are held in different positions and these contrasting architectures were mimicked by the fluorescent probes. The resulting chimeras recapitulated the original proteins localization, indicating that ALPS motifs are sufficient to specifically localize proteins. Modulating the electrostatic or hydrophobic content of Nup133 ALPS motif modified its avidity for cellular membranes but did not change its organelle targeting properties. In contrast, the structure of the backbone surrounding the helix strongly influenced targeting. In particular, introducing an artificial coiled-coil between ALPS and the fluorescent protein increased membrane curvature sensitivity. This coiled-coil domain also provided membrane curvature sensitivity to the amphipathic helix of Sar1. The degree of curvature sensitivity within the coiled-coil context remains correlated to the natural curvature sensitivity of the helices. This suggests that the chemistry of ALPS motifs is a key parameter for membrane curvature sensitivity, which can be further modulated by the surrounding protein backbone.

## Introduction

Eukaryotic cells are compartmentalized into organelles fulfilling specialized functions. Organelles are delineated by lipid bilayers of specific composition and structure [1]. They exhibit a variety of shapes such as spherical lysosomes and peroxisomes, the intricate network of tubules and cisternae of the endoplasmic reticulum (ER) or stacks of fenestrated cisternae surrounded by small transport vesicles in the Golgi apparatus. Each compartment is thus characterized by a combination of high and low curvature regions: high curvature is found in ER tubules, at the edges of ER or Golgi cisternae and in trafficking vesicles, while the nuclear envelope (NE), ER flat sheets and the Golgi stacks have a low curvature. Saddle-like topologies, a combination of positive and negative curvature, are also found at nuclear pore complexes (NPCs) or at the neck of budding vesicles.

Membrane curvature can be generated by four distinct mechanisms [2,3]: (i) mechanical forces exerted by molecular motors, polymerizing microtubules or actin filaments, (ii) “scaffolding” by proteins or protein oligomers exhibiting a curved and cationic surface [4], (iii) insertion of a hydrophobic domain, acting as a wedge in the lipid bilayer and inducing local curvature [5] and (iv) molecular crowding, where the lateral pressure induced by high protein density at the membrane surface is released by membrane curvature [6,7].

In addition to their role as physical barriers between compartments, curved membranes constitute targeting signals, sensed by specialized protein domains. This allows recruitment of molecules with acute precision in space and time. Membrane curvature sensing can be mediated by geometry: a convex and cationic protein surface senses a concave and anionic bilayer. A well-characterized example is the BAR domain, an elongated crescent-shaped dimer [8]. Membrane curvature sensing can also be achieved by amphipathic helices [9,10], like ALPS motifs (Amphipathic Lipid Packing Sensors) [11–13]. ALPS motifs are 20–40 amino acids peptides found in proteins targeted to curved regions of organelles. They are unfolded in solution and adopt an amphipathic helix (AH) conformation in the presence of curved membranes. Despite a lack of sequence homology, they share some common characteristics [13,14]. Compared to canonic AHs, they contain few charges and a polar face mainly composed of Glycine, Serine and Threonine residues. Of note, ALPS motifs are mostly targeted to membranes with low levels of negatively charged lipids, *i*.*e*. the early secretory pathway including the ER network and the *cis*-Golgi. Due to the lack of charges, ALPS motifs rely mainly on hydrophobic interactions to partition into the bilayer. This calls for a good accessibility of the phospholipid aliphatic chains, which is typically the case in convex membranes or in loosely packed bilayers. Supporting this model, adding charges at the interface between the polar and hydrophobic faces of an ALPS motif diminishes its membrane curvature sensitivity [13]. Molecular dynamics (MD) simulations also suggest that hydrophobic residues of the ALPS motifs insert sequentially in pre-existing packing defects of the lipid bilayer [15]. This iterative binding process reconciles the increased membrane affinity of ALPS motifs for curved membranes [13] and a general model in which membrane curvature sensing is driven by a higher density of binding sites in curved membranes [16].

Interestingly, the mechanisms at play for membrane curvature sensing (geometric domains or AHs) are reminiscent of membrane shaping mechanisms, underlining that the border between membrane shaping and sensing is not clear-cut [17]. In addition, different degrees of membrane curvature co-exist in cells but are targeted by specific proteins. This is in particular true for ALPS-containing proteins: for instance the nucleoporin Nup133 is targeted to the nuclear pore membrane (saddle topology)[18], while GMAP210 or ArfGAP1 are specific of Golgi-derived vesicles (spheres)[19–24]. Additional domains of the protein surrounding the ALPS motif may be responsible for the specific organelle localization. However, another possibility is that, in addition to a general propensity to sense membrane curvature, ALPS sequences contain specific features to recognize their target. In this case, targets may be discriminated owing to their acutely defined degree of curvature, or to other characteristics like lipid composition.

To elucidate these questions, we studied the cellular localization of ALPS motifs outside their natural protein context. Two ALPS motifs (GMAP210 and Nup133) and a non-ALPS AH (Sar1) were grafted on fluorescent probes mimicking the architecture of the original proteins. We showed that these constructs recapitulated organelle specificity and curvature sensitivity. This supports that the backbone sequences surrounding these AHs are not required for their selectivity. We then focused on ALPS(Nup133) and showed that its membrane curvature sensitivity is rather permissive to point mutations. In contrast, the architecture of the surrounding protein modulates membrane curvature sensitivity. In particular, the introduction of a dimeric coiled-coil domain increases membrane curvature sensitivity of Nup133 ALPS motif, and ascribes curvature sensitivity to the AH of Sar1.

## Results

### Specificity of amphipathic helices outside their protein context

**Fig 1:**
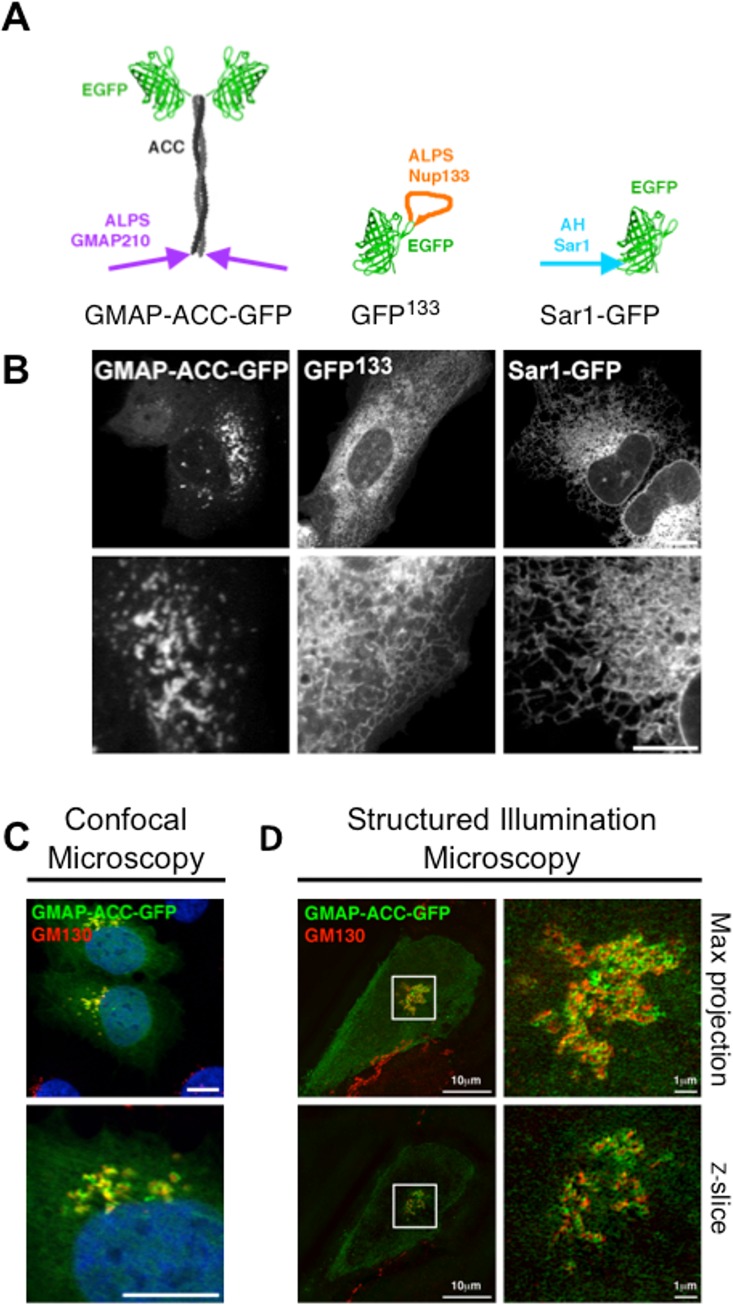
ALPS motifs confer organelle selectivity to a naïve protein. (A) Schematic of the GMAP-ACC-GFP, GFP^133^ and Sar1-GFP constructs. (B) Confocal images of live U2OS cells expressing GMAP-ACC-GFP, GFP^133^ or Sar1-GFP. (C-D) U2OS cells were transfected with GMAP-ACC-GFP, fixed and stained with the *cis*-Golgi marker anti-GM130. Cells were then imaged by confocal microscopy (C) or structured illumination (D). (D) Upper panels show a maximum projection of a Z-stack, lower panels are single cross-sections. Scale bars are 10μm unless otherwise stated.

Nup133 is an essential component of nuclear pore complexes (NPCs) [25–27]. We have previously shown that ALPS(Nup133) is required for its localization to the pore membrane and when taken away from its Nup133 context, the ALPS motif is localized at the ER [18]. The ALPS motif is located between 2 beta strands of the beta-propeller N-terminal domain of Nup133. As a result, its extremities are constrained at a distance of about 8Å ([28], S1A Fig). As this may influence its folding, we inserted the ALPS motif in a surface loop of EGFP to recapitulate the restraint (S1A Fig). On the other hand, GMAP210 is a golgin located at the *cis*-Golgi. Its C-terminal GRAB-domain interacts indirectly with Golgi stacks, while its N-terminal ALPS motif can capture vesicles involved in anterograde transport [29]. The two domains are separated by a long coiled-coil domain, responsible for protein parallel dimerization and its elongated, rod-like shape. Coiled-coils are present in other Golgi proteins, creating a dense matrix of elongated proteins around the Golgi stacks. As this may prevent accessibility of globular proteins to the Golgi network, this architecture was maintained in our chimera. An artificial dimeric coiled-coil was designed with a predicted length of about 12nm. It was inserted between the ALPS motif of GMAP210, positioned at its N-terminus, and EGFP (Fig 1A, S1B). We have shown previously that this construct localizes to trafficking vesicles, even in a naïve system [21,30]. Finally, we introduced in our study the N-terminal AH of Sar1, which is able to bend membranes *in vitro* [31]. Sar1 AH was grafted at the N-terminus of EGFP, with a short peptidic separator (S1C Fig).

The fluorescent chimeras (schematized in Fig 1A) were transiently transfected in U2OS cells and imaged live with a confocal microscope. While GFP^133^ and Sar1-GFP are localized at the ER (Fig 1B), GMAP-ACC-GFP is localized in the Golgi area (Fig 1B and 1C). As the NE is part of the ER network, it is striking that GFP^133^ keeps the ability to sense this membrane compartment. Thus, even when disconnected from their original protein environment, the three helices keep their organelle specificity. We then wondered if the fluorescent chimeras also recapitulated membrane curvature sensitivity in this cellular model.

**Fig 2:**
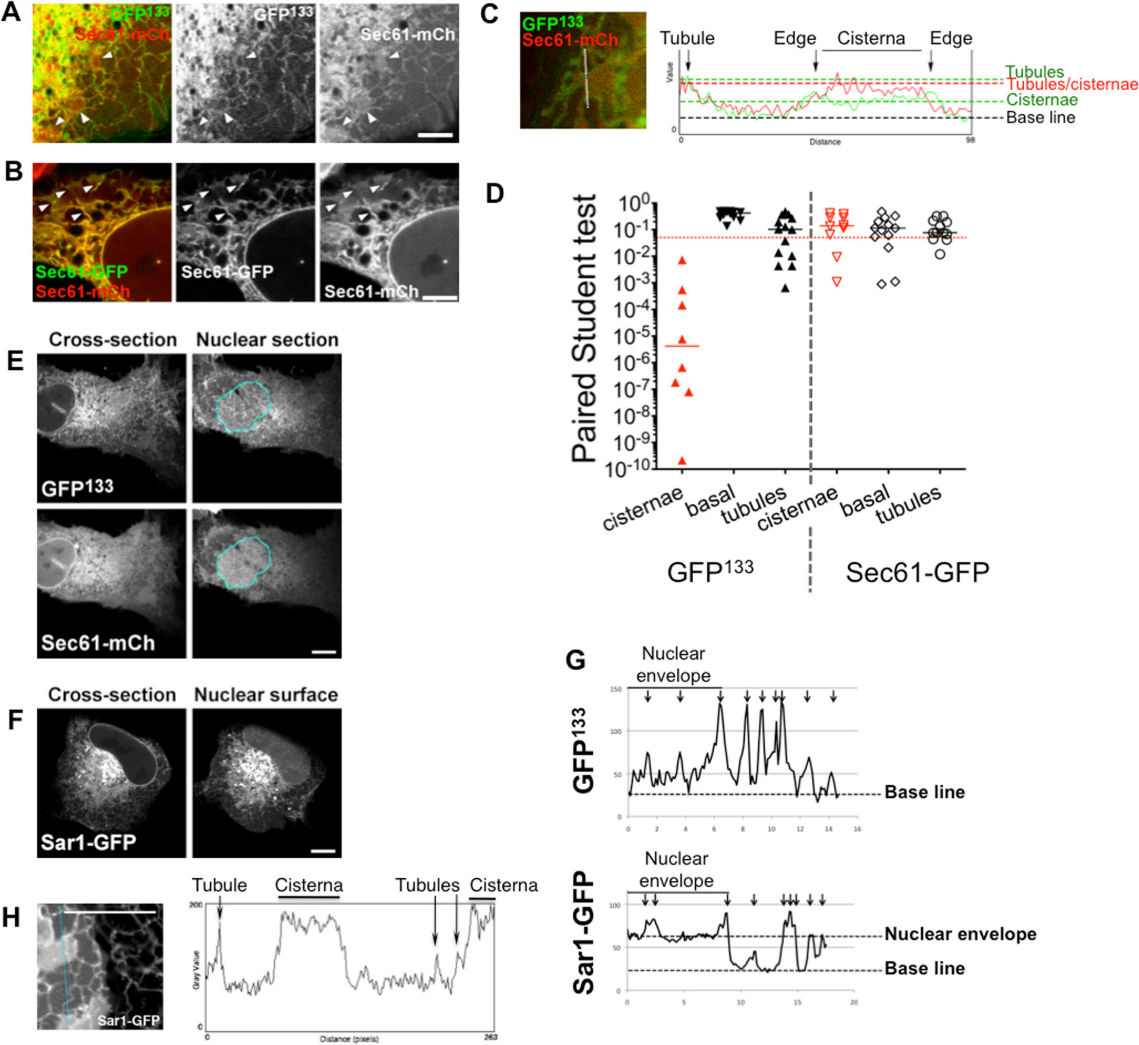
GFP^133^ and Sar1-GFP recapitulate membrane curvature sensitivity of native proteins. (A) Confocal image of a cell co-expressing GFP^133^ and the ER marker Sec61-mCherry. Arrowheads indicate flat cisternae. (B) Confocal image of a cell co-expressing Sec61-GFP and Sec61-mCherry. Arrowheads indicate flat cisternae. (C) Fluorescence intensities of GFP^133^ and Sec61-mCherry (right panel) along a line spanning an ER tubule and an ER flat sheet (in yellow in the left panel). (D) Several plots as in (C) were analysed in cells co-expressing GFP^133^ and Sec61-mCherry or Sec61-GFP and Sec61-mCherry. For each plot, paired Student t-tests were run to assess if the GFP and mCherry intensities are significantly different. This analysis was done on plot portions spanning cytoplasm (base line), cisternae or tubules. The dashed red line indicates a p-value of 0.05. Black lines represent median values. (E) Cross-section (left) and nuclear surface (right) of a cell co-expressing GFP^133^ and Sec61-mCherry. The cyan line delineates the nuclear surface. (F) Cross-section (left) and nuclear surface (right) of a cell expressing Sar1-GFP. (G) Representative intensity plots of GFP^133^ (upper panel) or Sar1-GFP (lower panel) along a line spanning a portion of nuclear envelope and a portion of cytoplasm. (H) Fluorescence intensity of Sar1-GFP (right panel) along a line spanning ER tubules and an ER flat sheets (in cyan in the left panel). Scale bars are 10μm unless otherwise stated.

As shown in Fig 1C, GMAP-ACC-GFP surrounds and only partially co-localizes with the *cis*-Golgi marker GM130. This localization reminds of the endogenous N-terminal part of GMAP210 [32] and suggests that the probe is associated with trafficking vesicles. This is even clearer when cells are imaged at higher resolution using structured illumination microscopy (Fig 1D). Single stack images (lower panel in Fig 1D) show very little co-localization between the *cis*-Golgi marker and GMAP-ACC-GFP, suggesting that the chimera surrounds the *cis*-Golgi cisternae. Regarding Nup133, nascent nuclear pores are rare events at the nuclear surface [33] and the ER density is high at the nuclear periphery. As an important fraction of GFP^133^ is present in the ER, the signal to noise ratio was too low to determine if a fraction of GFP^133^ targets nascent pores. Still we tested if GFP^133^ is a membrane curvature sensor within the ER network. We co-expressed it with Sec61-mCherry, an ER marker that equally stains ER tubules, cisternae and the NE. Images of flat ER patches at the cellular periphery show that GFP^133^ is barely detectable in cisternae, while Sec61-mCh equally stains tubules and cisternae, as expected (Fig 2A). In contrast, when Sec61-GFP and Sec61-mCherry are co-expressed, they completely overlay (Fig 2B). Intensity plots of GFP^133^ and Sec61-mCherry recorded along a line spanning a tubule and a cisterna (Fig 2C) confirm lower levels of GFP^133^ in cisternae. To check if this difference is significant, we analysed several intensity plots and ran paired Student t-tests between GFP and mCherry levels within cytoplasm (base line), cisternae or tubules (Fig 2D). The only significant difference was found for GFP^133^ *vs* Sec61-mCherry in ER cisternae.

In confocal sections, GFP^133^ seemingly stains the NE, which is a continuous network of flat membranes, only interrupted by NPCs (Fig 2E, left panels). As this may be due to the high density and close vicinity of the ER network, we imaged the basal surface of nuclei by confocal microscopy (delineated in cyan, Fig 2E, right panels): while Sec61-mCherry localization is rather homogenous, indicating its binding to the flat NE, GFP^133^ exhibits a reticulated staining, supporting that the probe is localized in neighbouring ER tubules. In comparison, Sar1-GFP uniformly stains the flat NE (Fig 2F), and both tubules and peripheral ER cisternae (Fig 2H). To better illustrate these differences, representative intensity plots of GFP^133^ or Sar1-GFP along a line spanning a portion of NE and a portion of cytoplasm are shown in Fig 2G. There is a significant amount of Sar1-GFP at the NE surface while GFP^133^ levels in the NE are close to basal levels, except for a few adjacent tubules indicated by arrows. This further confirms that the GFP^133^ chimera recapitulates Nup133 membrane curvature sensitivity, in contrast with Sar1-GFP, equally present in ER tubules and flat sheets.

Altogether, these data show that, when displaced from their natural host protein to a similar protein environment, the three AHs tested in this study conserve their properties towards both membrane curvature and organelle specificity. The determinants underlying these properties likely reside in the ALPS motif / AH sequence itself. In the absence of *bona fide* consensus ALPS sequence, we hypothesized the peculiar behaviours of the helices reside in their general physico-chemical properties.

Shown in Table 1 is a comparison between ALPS motifs and AHs targeted to distinct organelles of the early secretory pathway. Target membranes can be ordered from lower to higher curvature degree as follows: ER cisternae < NPCs < ER tubules < trafficking vesicles (see also S1D Fig). ER subcompartments exhibit a lower curvature degree, and thus less packing defects [34], than COP vesicles. This raises the possibility that ALPS specificity is due to acute sensitivity to membrane curvature degree rather than compartment recognition. As seen in Table 1, hydrophobic residues density is lower in membrane curvature sensors than in classical AHs (not sensitive to curvature, [35–37]). How AHs can discriminate between flat and curved membranes is not formally demonstrated, but our model is that such helices are too weak to bind flat membranes. Such a weakness can be due to lack of charges, as for ArfGAP1 [13] or low hydrophobicity, as for alpha-synuclein [21]. Modulating the hydrophobic content of AHs could change their targeting. In this respect, the ALPS motif of Nup133 is a target of choice as it senses membranes exhibiting an “intermediate” curvature degree (Table 1), and we can try to increase or lower its curvature sensitivity.

**Table 1:** Comparison of ALPS motifs and canonic amphipathic helices.

	Protein name	AH sequence	Position in protein	Length of AH	Number of charges	Hydrophobic density (% of hydrophobic residues)	Target	Topology of membrane target	Relative degree of curvature (compared to ER tubules)	References
**Golgi ALPS**	**GMAP210**	_1_ **M**SS**WL**GG**L**GSG**L**GQS**L**GQ**V**GGS**L**QS**L**TGQ**L**SN**F**TKD**ML**_38_	N-terminal + coiled coil	38 AA	2	34.2	COPII vesicles	Spheres, 25nm radius	+2	[13,15,21,29,32]
	**ArfGAP1**	_**199**_ **FL**NSA**M**SS**LY**SG**W**SS**F**TTGASK**F**AS_223_	Internal	25 AA	1	32	COPI vesicles	Spheres, 25nm radius	+2	[12,15,22]
**ER ALPS**	**Barkor**	_471_GG**MI**SSAAAS**V**TS**WF**KA**Y**TG_490_	Internal	20 AA	1	30	autophagosomes	?	?	[46]
	**Kes1**	_7_SSS**W**TS**FL**KS**L**AS**F**NGD**L**SS**L**SA_29_	N-terminal	23 AA	2	30.4	ER tubules?	Cylinder, 25nm radius	+1	[13,47]
	**Nup133**	_245_ **L**PQGQG**ML**SG**L**GRK**V**SS**LF**G**IL**S_267_	Internal	23 AA	2	39.1	Nuclear Pore Complex	Saddle: negative curvature 75nm radius, positive curvature 25nm radius	+2/3	[13,18]
**Bending Ahs**	**Arf1**	_1_ **M**GN**IF**AN**LF**KG**LF**G_14_	N-terminal	14 AA	3	50	*cis*-Golgi	Flat membrane (*cis*-Golgi)	0	[35]
	**ySar1**	_1_ **M**AG**W**D**IF**G**WF**RD**VL**AS**L**G**LW**NKH_23_	N-terminal	23 AA	4	47.8	ER	Flat ER cisternae	0	[31]
	**Yop1**	_136_AR**IIY**QK**IV**AP**L**_149_	Internal	14 AA	3	42.9	ER	Tubulates flat ER cisternae	0	[36]
	**Atlastin**	_478_ **F**GGK**L**DD**F**AT**LLW**EK**FM**R_496_	C-terminal	19 AA	6	42.1	ER	ER	?	[37]

### Influence of hydrophobicity

**Fig 3:**
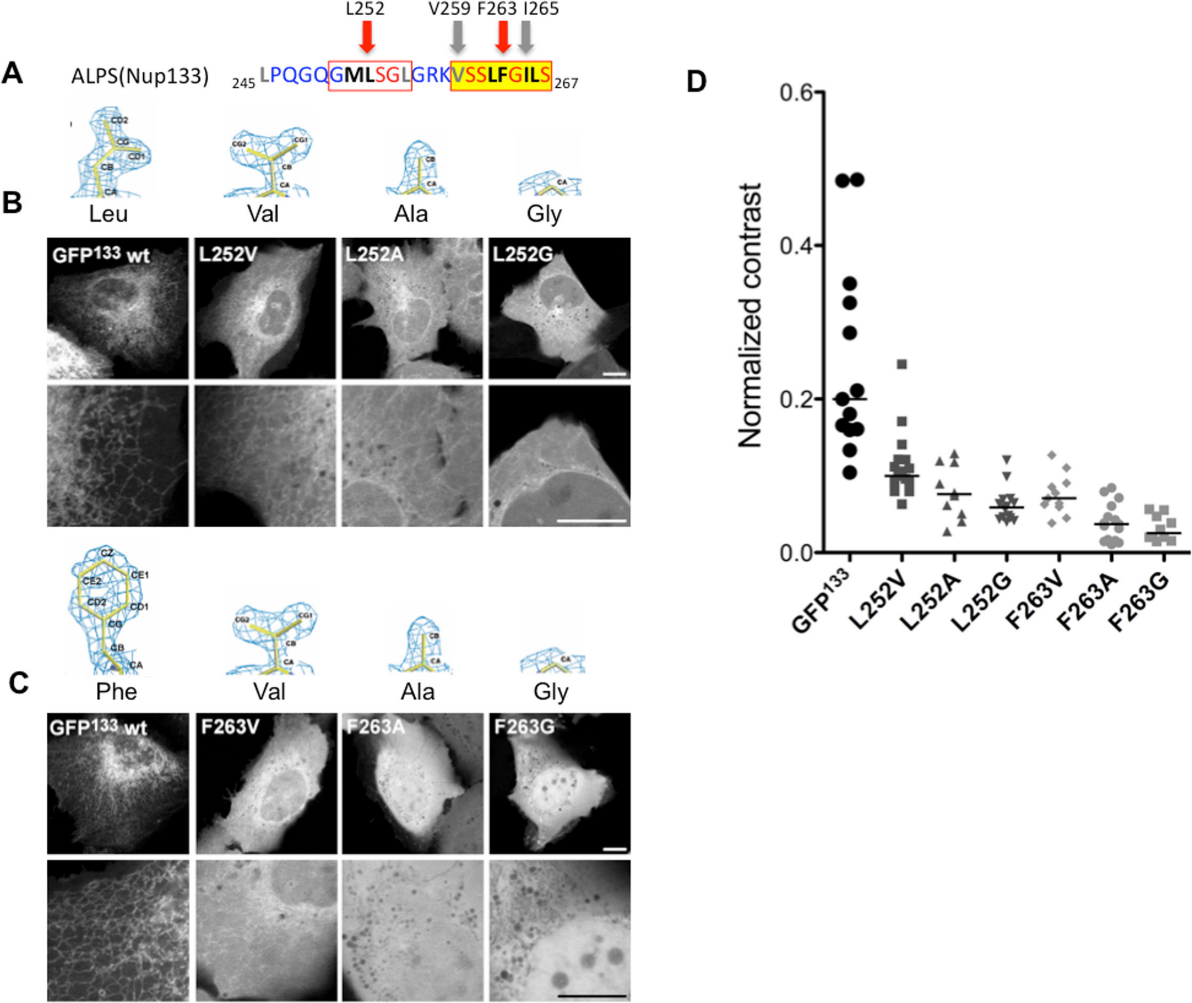
Reducing its hydrophobicity does not increase membrane curvature sensitivity of Nup133 ALPS motif. (A) Amino-acid sequence of the ALPS motif of Nup133. The strongest hydrophobic region is highlighted in yellow, and the secondary region is framed in red. The hydrophobic residues mutated in this study are indicated by arrows. (B) L252 in GFP^133^ was mutated to less hydrophobic residues, namely Valine, Alanine and Glycine. (C) F263 in GFP^133^ was mutated to less hydrophobic residues, namely Valine, Alanine and Glycine. Scale bars are 10μm. (D) Normalized GLCM contrasts, as an indication of membrane-bound fractions, were measured in peripheral regions of cells transfected with the indicated constructs. Horizontal bars represent median values.

As depicted in Fig 3A, ALPS(Nup133) contains a moderately hydrophobic region around residues M251, L252, and a stronger region around the hydrophobic pairs [L262, F263] and [I265, L266] (AA numbers correspond to their position in the sequence of human Nup133). We mutated L252 or F263 into valine, alanine or glycine, gradually reducing the volume of hydrophobic side chains. These mutants were expressed and imaged in cells (Fig 3B and 3C). No obvious relocalization to the Golgi apparatus or other organelles was observed. However, the constructs became apparently more soluble as their hydrophobicity decreased. Importantly, all constructs have similar expression levels (S3F Fig), excluding that the soluble fraction is due to an excess of protein. Of note, decreased membrane binding is accompanied by protein relocation to the nucleus. In fact, the small size of GFP^133^ allows in principle its free diffusion to the nucleus [38], but this is likely prevented as long as it is sequestered in the cytoplasmic compartment due to ER binding. Based on nuclear localization, F263 mutants seem to have lower membrane binding, suggesting the stronger hydrophobic region is critical for GFP^133^ binding to membranes.

To compare membrane binding by the different constructs more quantitatively, we estimated the ratio between cytosolic and ER-bound protein. However, the complex shape of the ER makes it difficult to measure ER *vs* cytosol fluorescence intensities. Instead, we quantified how contrasted the probes localization is: a protein mostly localized to the ER renders a strongly contrasted fluorescence image, as compared to a soluble protein. To this end, we measured “normalized contrasts”, based on Haralick’s texture parameters [39], in areas of the peripheral ER (see Material and methods, and S2 Fig for details). Comparing the normalized contrasts of the constructs shows a gradual loss in membrane binding as the volume of hydrophobic residues is reduced, and confirms that F263 mutation has a stronger effect (Fig 3D). This suggests that, in the case of ALPS(Nup133), weakening its hydrophobicity is not sufficient to modify its membrane curvature sensitivity, although this effectively decreases membrane targeting.

**Fig 4:**
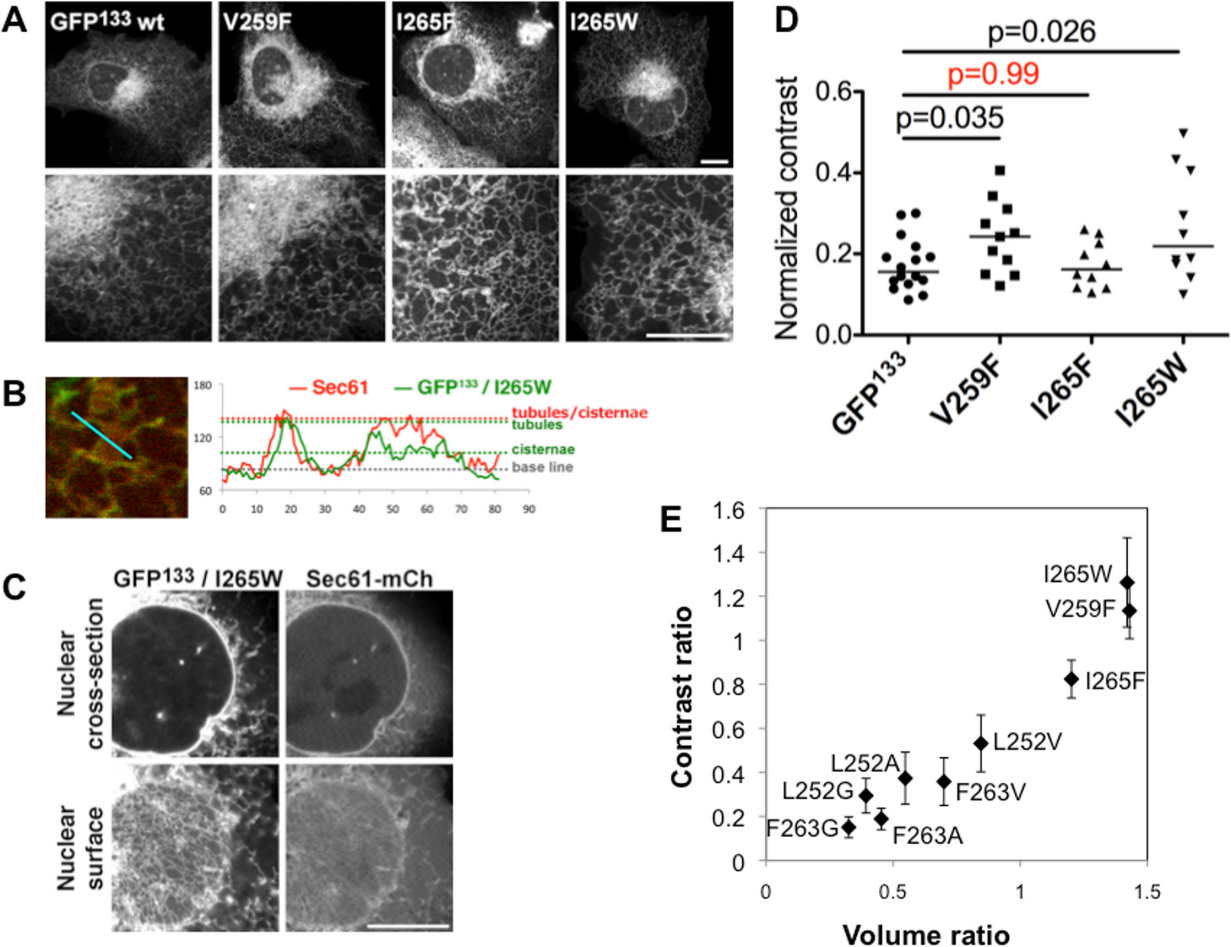
Increasing its hydrophobicity does not impair membrane curvature sensitivity of Nup133 ALPS motif. (A) V259 and I265 of GFP^133^ were mutated to bulkier hydrophobic residues. (B) Intensity plots of GFP^133^ / Sec61-mCherry along a line spanning an ER tubule and an ER flat sheet. (C) Cross-section and nuclear surface of a cell co-expressing GFP^133^ and Sec61-mCherry. All scale bars are 10μm. (D) Normalized GLCM contrasts measured in peripheral regions of cells transfected with the indicated constructs. Horizontal bars represent median values. (E) For each mutant, we calculated the side chain volume ratio of the mutated to wt residues, and the average of the corresponding normalized contrast ratios. Contrast ratios were then plotted as a function of volume ratios. Error bars are standard deviations.

Conversely, we reinforced some hydrophobic residues, replacing them by amino acids with large aromatic side chains. Valine 259 and Isoleucine 265 were mutated in Phenylalanine or Tryptophan, and the resulting constructs were expressed in U2OS cells (Fig 4A, S3G). The overall ER localization did not differ from the wild-type probe, except for V259W mutant that could not be expressed. When looking in more details, the mutants also exhibit a preference for ER tubules over cisternae (Figs 3B and S3A–S3D) and the apparent NE localization is due to the close proximity of ER tubules, as for the wild-type protein (Fig 4C). However, the reinforced hydrophobic mutants appear to be less soluble than the wild-type. This was confirmed by contrast measurements from areas in the peripheral ER of transfected cells (Fig 4D). Interestingly, only the I265F mutant was not significantly different from wt GFP^133^. As Isoleucine and Phenylalanine side chains have similar volumes (S3E Fig, [40]), we wondered if membrane binding could be directly correlated to the volume of hydrophobic side chains. When plotting the average normalized contrasts measured for our different constructs (weakened as well as reinforced mutants) over the change in side chain volume (ratio of mutated residue volume over wt residue), we observe a correlation between side chain volume of hydrophobic residues and membrane binding (Fig 4E). This supports that ALPS motif binding to membranes relies primarily on hydrophobic interactions. Most interestingly, the overall volume rather than the identity or position of the hydrophobic residue in the ALPS sequence is determinant for membrane binding. This agrees with our model obtained from MD simulations, where hydrophobic residues partition in the bilayer in a stochastic and sequential manner when they coincide with pre-existing packing defects [15].

### Role of charges

**Fig 5:**
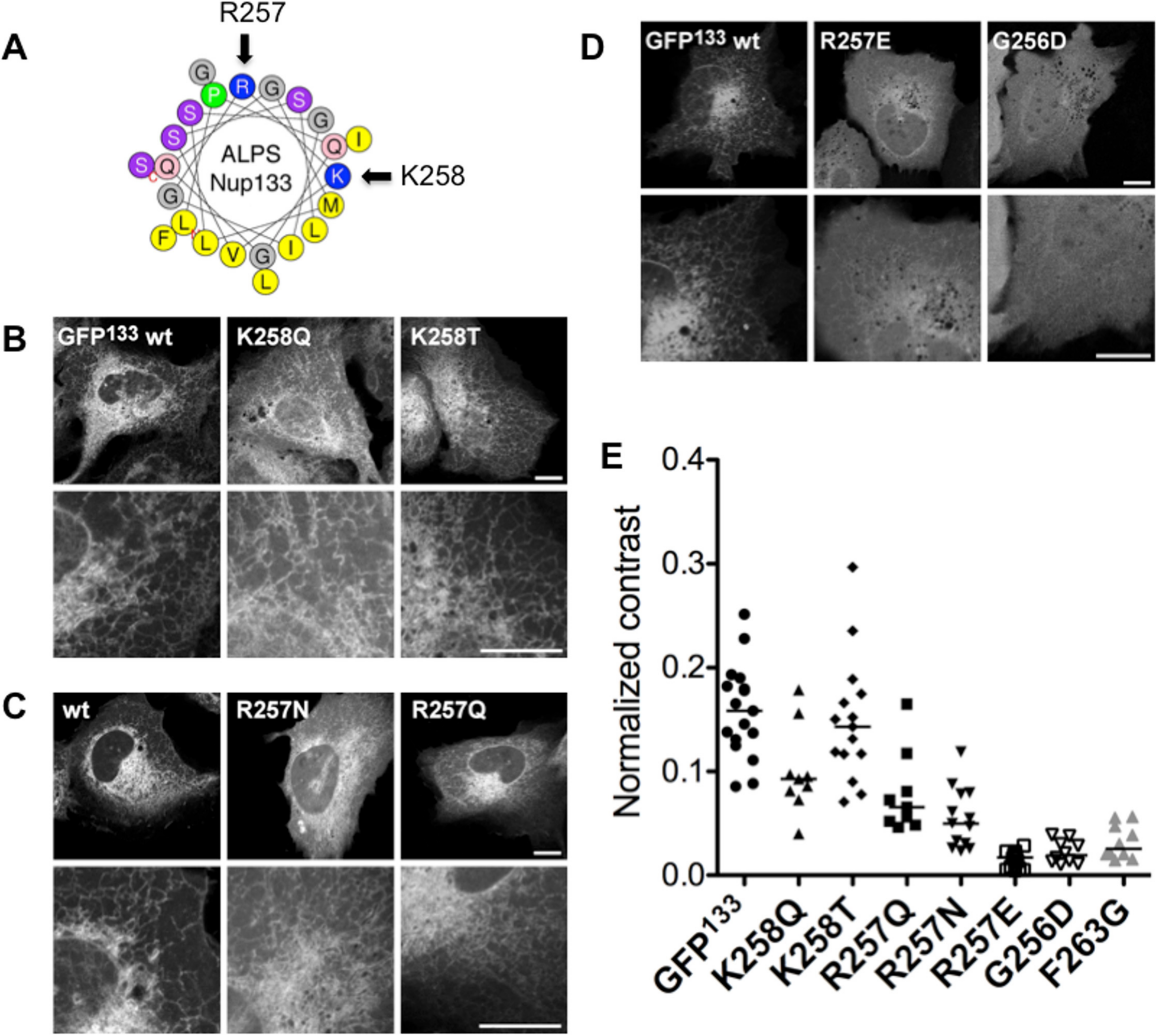
Altering the charges of ALPS Nup133 does not change its specificity. (A) Helical projection of Nup133 ALPS motif, showing the position of the two basic residues. The projection was generated by the Heliquest software (http://heliquest.ipmc.cnrs.fr). (B) Confocal images of live cells expressing GFP^133^ and mutants of lysine K258. (C) Confocal images of live cells expressing GFP^133^ and mutants where the R257 residue has been mutated to uncharged residues. (D) Neutralizing or adding a negative charge at the interface between the polar and hydrophobic faces of the helix reduces its binding to membranes. Scale bars = 10μm. (E) Normalized GLCM contrasts of mutated constructs, as indicated. Bars are median values.

ALPS motifs are weak electrostatic interactors: indeed, a major difference between ALPS motifs and classical AHs is the scarcity of charges in ALPS motifs, balanced by abundant Glycine and Serine or Threonine residues. Adding charges at the interface between the polar and hydrophobic faces of ArfGAP1 ALPS helix increased binding to flat membranes [13]. Interestingly, compared to other ALPS motifs, Nup133 contains two basic residues, which may decrease its curvature sensitivity. In particular, Lysine 258 lies at the interface between the hydrophobic and polar faces of the AH, according to the helical projection (Fig 5A). We mutated independently the two charges to test if decreasing electrostatics increases membrane curvature sensitivity. The basic residues were replaced by neutral amino acids with side chains of various lengths. This did not dramatically alter membrane curvature sensitivity, as no obvious recruitment to other organelles was observed (Fig 5B and 5C). However, reducing the charge of the ALPS motif reduced membrane binding (Fig 5B, 5C & 5E). In line with this, complete neutralization of the ALPS motif, by replacing the arginine by a glutamate, mostly solubilized the protein (Fig 5D and 5E). Again, the apparent solubility of the mutants is not due to increased expression levels (S3H Fig). This series of experiments suggest electrostatics participate in membrane binding of ALPS(Nup133). However, removal of either charge retains more ER binding than the reduced hydrophobic mutant F263G (Fig 5E). Only the neutral mutant is as soluble. This supports that electrostatics are not the main driving force in ALPS recruitment to curved membranes.

### Influence of the backbone surrounding the ALPS motif

**Fig 6:**
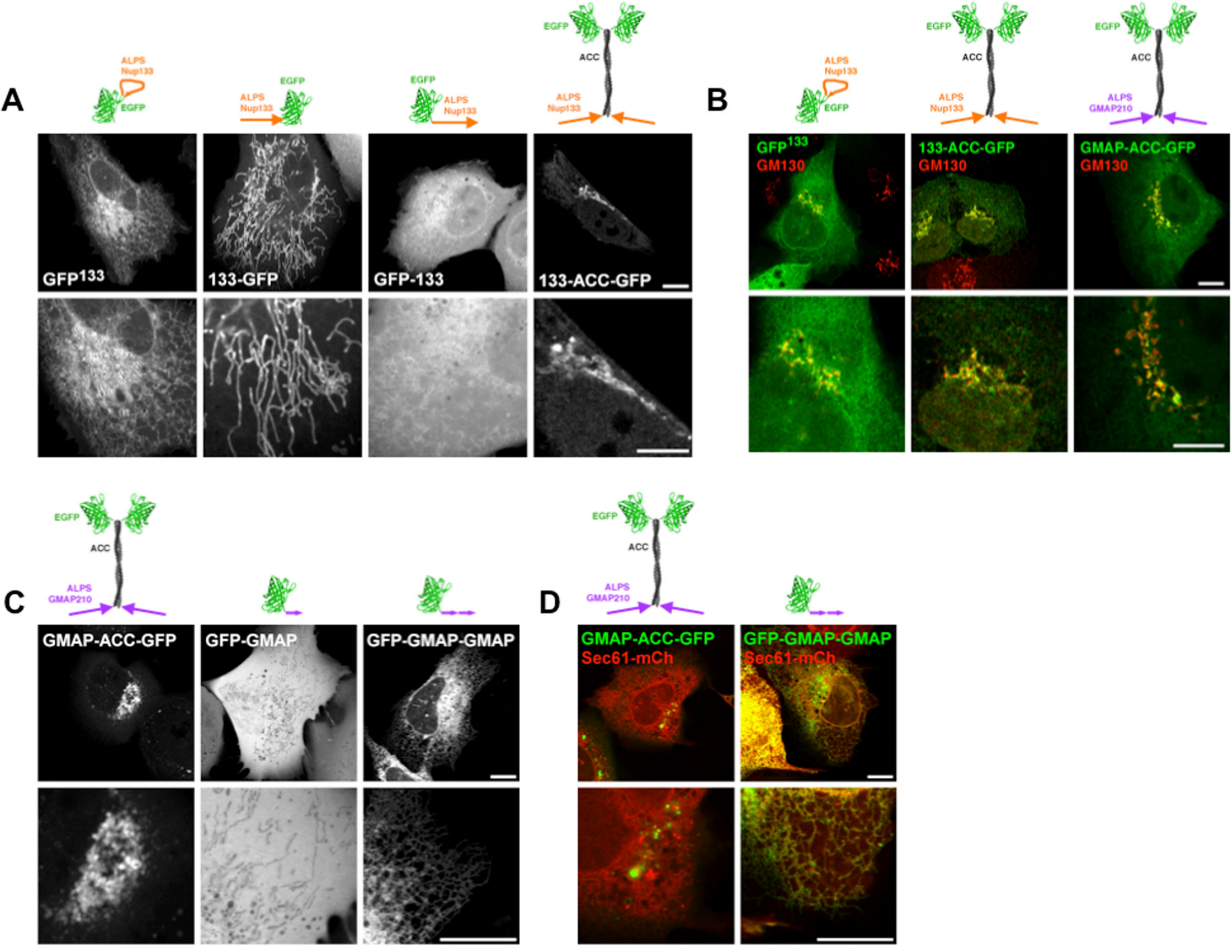
Geometry of the ALPS motif, imposed by the backbone, influences Nup133 ALPS motif localization. (A) Confocal images of live cells transfected with GFP^133^, 133-GFP, GFP-133 or 133-ACC-GFP. (B) Confocal images of cells transfected with GFP^133^, 133-ACC-GFP or GMAP-ACC-GFP, fixed and stained with anti-GM130 (red). Note that fixation alters ER morphology. (C) Confocal images of live cells transfected with GMAP-ACC-GFP, GFP-GMAP or GFP-GMAP-GMAP. (D) Confocal images of live cells co-transfected with Sec61-mCherry and GMAP-ACC-GFP or GFP-GMAP-GMAP. Scale bars = 10μm.

Beyond their different physico-chemical properties, the helices of Sar1, Nup133 and GMAP210 differ remarkably in the way they are exposed in their host proteins. Whereas GFP^133^ and Sar1-GFP are predicted to be monomeric globular proteins, GMAP-ACC-GFP is a long rod-like homo-dimer. Moreover, the ALPS motif of Nup133 is restrained, its extremities being kept at a fixed distance within the beta-propeller structure. As this constraint was conserved in the GFP^133^ chimera, it may be critical for localization. To test this, we created a constraint-free N-terminal fusion with EGFP. 133-GFP was dramatically relocated to mitochondria (Fig 6A, S4A). Indeed, ALPS(Nup133), with its alternation of hydrophobic and polar / basic residues, meets, when positioned at the N-terminus, the requirements for mitochondrial targeting [41,42] (43% prediction score according to the prediction software for mitochondrial target sequences Mitoprot II http://ihg.gsf.de/ihg/mitoprot.html). This phenomenon introduces a bias to analyze the role of conformational restraint on ALPS extremities in membrane curvature sensing. We thus constructed the C-terminal fusion GFP-133, which retains some ER localization, but is more soluble than the original construct (Fig 6A). This suggests that restraining the degrees of freedom to be explored by ALPS(Nup133) is optimal for membrane binding. This conformation may for instance favour helical folding of the ALPS peptide.

### Presence of the coiled-coil enhances membrane curvature sensitivity

We then introduced ALPS(Nup133) in the same construct as ALPS(GMAP210). 133-ACC-GFP was dramatically relocalized to the *cis*-Golgi (Fig 6A and 6B). Similarly to GMAP210, co-localization with the *cis*-Golgi marker GM130 was partial (Fig 6B), suggesting that 133-ACC-GFP is targeted to trafficking vesicles rather than Golgi cisternae. Importantly, the chimera is not targeted to mitochondria (S4A Fig), nor alters their morphology.

Conversely, we tested the influence of the coiled-coil on GMAP210 localization. When expressed in U2OS cells, the C-terminal fusion GFP-GMAP was soluble (Fig 6C). This could be due to insufficient membrane avidity of the monomeric ALPS compared to the GMAP-ACC-GFP dimer. We then duplicated ALPS(GMAP) in the C-terminal fusion with GFP and the resulting construct (GFP-GMAP-GMAP) localized to the ER (Fig 6C and 6D), with a fraction still localized at the Golgi. This supports that i) two ALPS motives are required for membrane binding and ii) the coiled-coil indeed favours Golgi targeting. In a previous study, Cardenas and collaborators showed that a GMAP-GFP construct is indeed more soluble when the coiled-coil is removed and membrane binding was increased in a GMAP-GMAP-GFP construct. However GMAP-GFP remains partially localized at the Golgi in their study. This may be due to the N-terminal position of the ALPS motif or to the cell type they use (RPE1 cells). Indeed, we have noticed that these cells have a very low amount of ER tubules compared to ER cisternae (unpublished observations). Importantly, the GFP-GMAP-GMAP construct still retains membrane curvature sensitivity, as illustrated by the lack of targeting to the flat NE (S4B Fig).

As the coiled-coil used in our constructs was artificially designed, it is unlikely to interact with endogenous partners. Moreover, in the absence of AH, ACC-GFP is exclusively soluble (S5A Fig). In addition, when the highly cationic AH of the yeast protein Spo20 is grafted to the ACC-GFP backbone, the resulting construct does not localize to the Golgi, but at the negatively charged plasma membrane [30]. Altogether, these arguments rule out a direct targeting of the ACC to the Golgi / trafficking vesicles. We rather favour that the coiled-coil structurally rearranges the ALPS motifs and thus modifies its targeting specificity. Importantly, a number of proteins localized at the Golgi network also possess coiled-coils. As a consequence, the Golgi apparatus is surrounded by a dense matrix of elongated proteins and this may prevent accessibility to globular proteins [43]. The coiled-coil we designed is predicted to be 12nm long [44], keeping the globular EGFP away from the membranes. To test if the rod-like geometry of the coiled-coil is indeed an asset for Golgi localization, we shortened it. However, the resulting GMAP-shortACC-GFP exhibited the same localization as the original probe (S5B Fig). As the shortened ACC is predicted to be 4nm long, about the size of EGFP itself and the thickness of the bilayer, the resulting protein is rather globular, suggesting an elongated shape is not required to access the Golgi apparatus.

**Fig 7:**
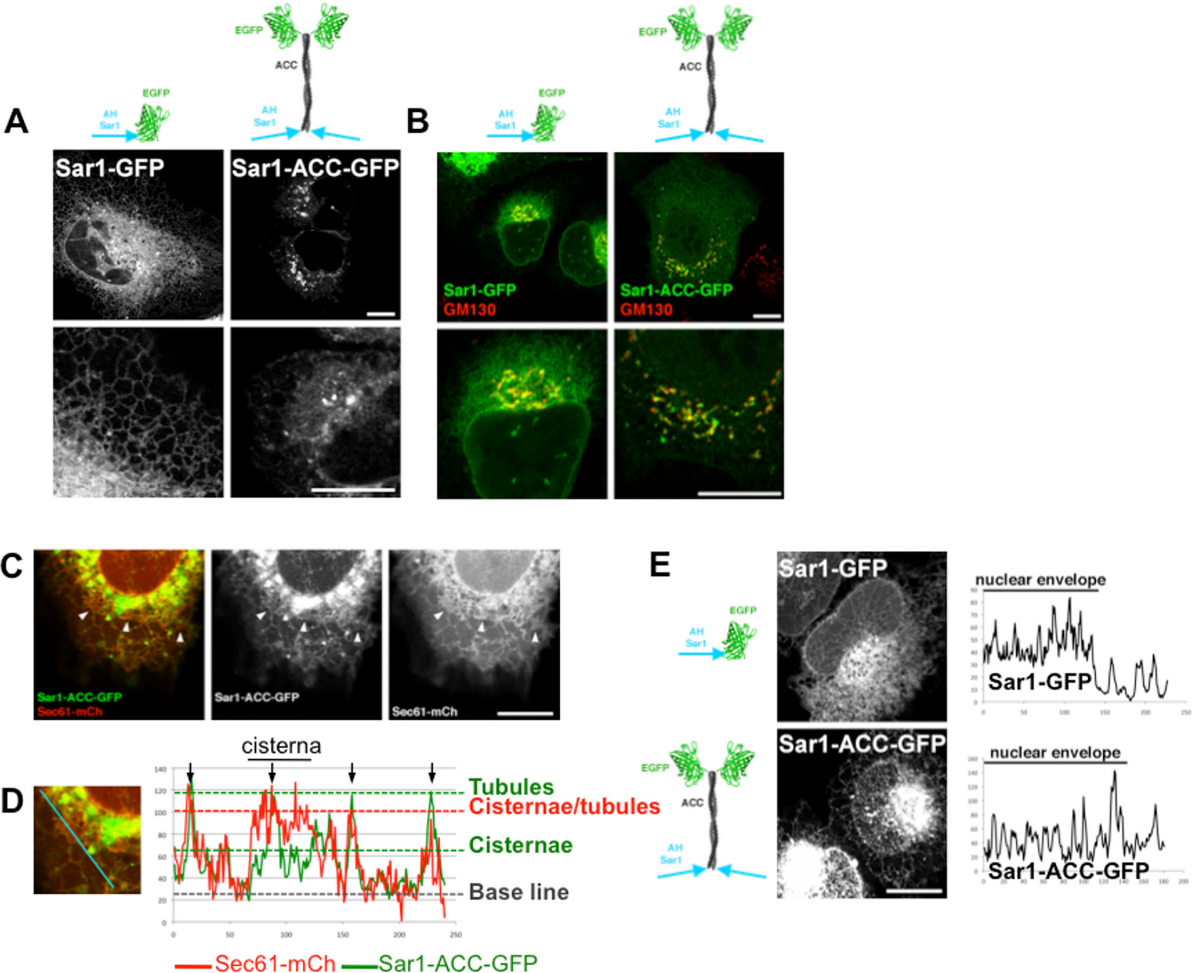
The coiled-coil domain provides membrane curvature sensitivity to Sar1 AH. (A) Confocal images of live cells transfected with Sar1-GFP or Sar1-ACC-GFP. (B) Confocal images of cells transfected with Sar1-GFP or Sar1-ACC-GFP, fixed and stained with anti-GM130 (red). (C) Confocal image of a cell co-transfected with Sar1-ACC-GFP and Sec61-mCherry. Arrowheads indicate ER cisternae. (D) Relative intensities of Sar1-ACC-GFP and Sec61-mCherry along a line spanning ER tubules and a cisternae (shown in cyan in left panel). (E) Confocal images of the nuclear surface of cells expressing Sar1-GFP or Sar1-ACC-GFP. On the right are shown representative intensity plots along a segment spanning portions of nuclear envelope and cytoplasm. Scale bars = 10μm.

Finally, we wondered if the artificial coiled-coil could also retarget a non-ALPS AH. We thus grafted Sar1 AH to ACC-GFP. This dramatically relocalized Sar1 AH to the cis-Golgi (Fig 7A and 7B). Again, the partial co-localization of Sar1-ACC-GFP with GM130 suggests that the construct is targeted to trafficking vesicles. However, at higher exposure, it is clear that a fraction of the protein localizes at the ER (Fig 7C and 7E). But in contrast with Sar1-GFP, Sar1-ACC-GFP selectively targets ER tubules (Fig 7C and 7D), suggesting that it gained membrane curvature sensitivity. In line with this, the construct is excluded from the flat NE (Fig 7E). Altogether, these experiments support that addition of the artificial coiled-coil to the three helices we tested provides a gain in membrane curvature sensitivity.

## Discussion

The aim of this study was to understand the molecular determinants underlying membrane curvature sensing by ALPS motifs. In particular, we wondered how distinct curved membranes of the early secretory pathway are discriminated by proteins bearing different ALPS motifs. We wanted to elucidate i) if determinants driving specific targeting lie solely in the ALPS motif or if the surrounding protein backbone is involved, ii) if organelle recognition is driven by organelle identity (lipid or protein composition) or by subtleties in their curvature degree, and iii) we wanted to identify the molecular determinants in ALPS sequences supporting their specificity towards organelles. To address the multiple aspects as of target membranes (shapes, composition…), we conducted this study in cultured cells. U2OS cells were chosen for their morphology, allowing to visualize the tubular ER network and some flat ER patches at the cell periphery. The use of appropriate organelle markers combined with confocal and structured illumination microscopy made it possible to discriminate membrane compartments.

We showed that the ALPS motifs of GMAP210 and Nup133 and the AH of Sar1, when taken away from their original surrounding backbone and put back in a construct that mimics the original protein architecture, are sufficient to target a chimeric fluorescent construct with specificity towards membrane curvature and organelle (Figs 1 & 2). This is especially remarkable since ALPS motifs are short peptides (20–40 AA) with no obvious sequence conservation. Focusing on the ALPS motif of Nup133, we then explored the key features in its sequence responsible for specificity. A current model to explain membrane curvature sensitivity by AHs states that a strong imbalance between their polar and hydrophobic faces creates a weakness regarding electrostatic or hydrophobic interactions (discussed in [21,45]). This prevents their binding to flat membranes and makes them sensitive to membrane curvature. Hence the idea that the weaker (less hydrophobic / less charged) the AH, the more sensitive to membrane curvature it is. This model correlates well with the analysis of various AHs presented in Table 1: the AH density of hydrophobic residues is inversely proportional to the curvature degree of its targets, suggesting that the more hydrophobic, the less sensitive to membrane curvature. We thus decided to modulate the hydrophobic content of ALPS(Nup133) and assess its membrane curvature sensitivity. However, all point mutations attempted on GFP^133^ only decreased membrane binding, with no obvious change in selectivity. Nup133 ability to sense curved membranes is thus permissive to point mutations. Interestingly, the hydrophobic residues L252, V259, F263 and I265, mutated in our study, are not identically conserved among species. Since membrane curvature sensing is essential for NPC assembly during interphase [18], this permissiveness may have contributed to the maintenance of a crucial function across evolution.

Again, aiming at weakening ALPS(Nup133), we gradually decreased its cationic charge. This did not increase its membrane curvature sensitivity either, but only affected membrane binding. Interestingly, the ALPS motif of Barkor/Atg14 is also insensitive to charge removal [46].

In contrast with this resistance to chemical changes, the way ALPS motifs are held in their surrounding backbone dramatically impacts their selectivity. ALPS(Nup133) is located in the beta-propeller domain of Nup133, constrained between two beta-sheets [28]. Most of the ALPS motif was not resolved in the crystal structure, supporting a loose conformation in the absence of membrane interaction. However, the rigid scaffold provided by the beta-propeller creates a constraint between the extremities of the ALPS motif, distant of about 8Å according to the crystal structure. We recapitulated this peculiar conformation in GFP^133^ and this chimera mimicked both organelle selectivity and membrane curvature sensitivity. In the absence of restraint, the specific ER tubule location was partially lost. As the predicted ALPS helix (about 35 Å) is longer than the distance between its extremities, the ALPS helix has to be broken, either by a flexible portion, or a kink. This conformation may facilitate helical folding or cooperative binding of several helical domains to curved membranes. Remarkably, ALPS motifs are presented in very different ways: besides GMAP210 and Nup133 characteristic displays, the ALPS motif of Kes1/Osh4 is N-terminal [47], the ALPS motif of Barkor/Atg14 is C-terminal [46] while the ALPS motifs of ArfGAP1, Vps41 or Synapsin are internal, but with no obvious structural restraint described so far [11,22,46,48]. The way ALPS motifs are presented to membranes may thus contribute to their specific localization.

**Fig 8:**
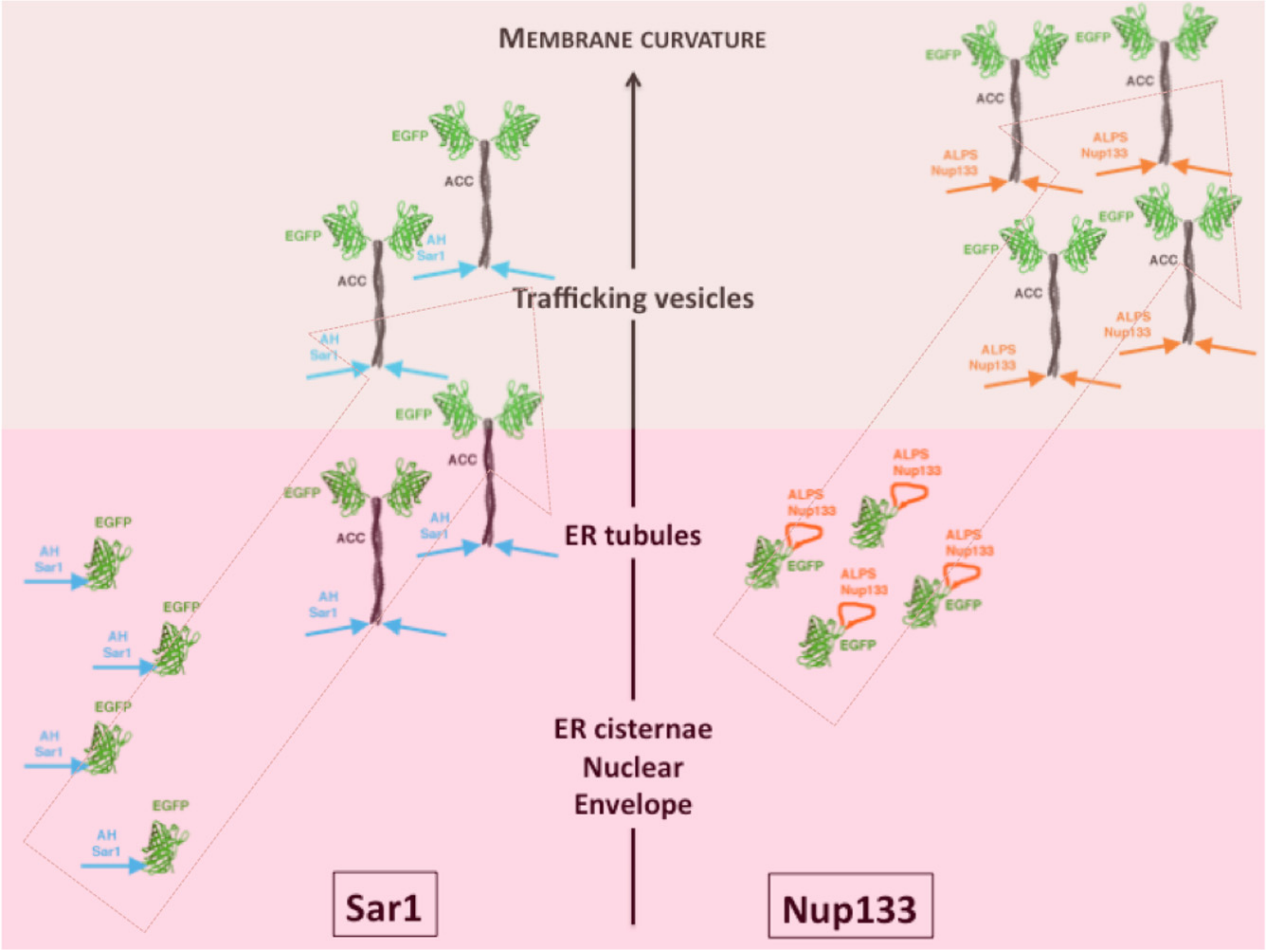
Schematic cellular localization of Sar1 AH and Nup133 ALPS motif within various backbones. This diagram illustrates that addition of the ACC domain increases membrane curvature sensitivity of the considered helices. But importantly, this gain remains correlated to their initial degree of membrane curvature sensitivity. This supports that the AH physico-chemical properties are determinant for membrane curvature sensitivity even when they are modulated by the surrounding backbone.

In line with this hypothesis, adding an artificial coiled-coil between the helix and the fluorescent probe targeted the fusion protein to the *cis*-Golgi area (Fig 6A and 6B). The partial co-localization with a *cis*-Golgi marker, very similar to that observed when GMAP-ACC-GFP is expressed, suggests 133-ACC-GFP is targeted to trafficking vesicles. A similar phenotype was observed with Sar1 AH. This raises several questions: i) how does the coiled-coil influence targeting? ii) is targeting driven by membrane composition or shape? Regarding the latter, the comparison between the ALPS motif of Nup133 and Sar1 AH is informative as both helices are targeted to the ER in their endogenous environment. When presented at the tip of an artificial coiled-coil, both constructs are localized to the *cis*-Golgi, but Sar1-ACC-GFP retains some ER localization. This supports that targeting is not dictated by membrane identity since both Golgi and ER are targeted. As the ER fraction of Sar1-ACC-GFP preferentially decorated tubules (Fig 7C and 7D), it suggests Sar1 AH gained membrane curvature sensitivity in the coiled-coil context. Interestingly, Sar1-ACC-GFP binds less curved membranes than 133-ACC-GFP and GMAP-ACC-GFP. The gradual sensitivity to membrane curvature is reminiscent of the relative sensitivity of the initial helices (see Fig 8). This indicates membrane curvature sensing depends both on intrinsic properties of the AHs and the surrounding protein architecture.

Then, how does the ACC act? As it was artificially designed, the coiled-coil itself is unlikely to interact with cellular proteins or lipid bilayers. Accordingly, ACC-GFP is soluble. Sar1-ACC-GFP and 133-ACC-GFP having somewhat different localizations also suggests that chimeras localization is not due to direct interaction of the ACC to target membranes. In line with this, when the non-related, highly cationic Spo20 AH is grafted to the ACC-GFP construct, it is localized at the negatively charged plasma membrane [30].

As many golgins harbour a coiled-coil, we first wondered if this structural feature could favour Golgi localization. Indeed, the Golgi apparatus is surrounded by a dense meshwork called the Golgi matrix, which may prevent access to bulky globular proteins and filter in elongated proteins. In line with this, a shorter GMAP210 is unable to tether vesicles around the Golgi; this is not due to a defect in vesicle binding, but rather to inability of bulky vesicles to enter the Golgi matrix to reach the shorter GMAP210 [20]. In our study, shortening the coiled-coil to render a more globular architecture while maintaining the dimer did not affect the peri-Golgi localization, showing that globular proteins can weave in the Golgi matrix, given that they are much smaller than vesicles.

Since Sar1 AH has the ability to bind flat membranes *in vivo* and *in vitro* (Fig 2H and [31]), our data suggest that the ACC-GFP backbone hinders binding to flat membranes. A possible explanation is that dimerization through the coiled-coil introduces a geometric constraint between the two AHs, which could be incompatible with binding to flat membrane. When designing the ACC-GFP construct, we assumed the linker is mostly flexible and unfolded, but it may actually have a propensity to fold as a helix and generate an angle between the coiled-coil and the N-terminal helix as illustrated in S6 Fig. More generally, dimerization constitutes an interesting asset to adapt to curved membranes as the relative orientation and distance between two membrane binding motifs can create a three-dimensional structure to hug the curved membrane. Playing on the relative position of monomers within a dimer can widen the range of membrane shapes that can be sensed. An example of such mechanism is the BAR protein mPACSIN: its structure reveals that a hinge movement between the two monomers can modulate the radius of the crescent [49]. This structural plasticity suggests that an array of membrane curvature degrees can be sensed by a single adaptable BAR domain. This also illustrates how dimerization can be an asset to optimize the geometric match between sensors and highly curved membranes. In the case of ALPS motifs, the coarse properties of the AH play a prominent role in membrane curvature sensing. But modulating ALPS properties by the way they are displayed to membranes broadens the range of curvature degrees that can be sensed and potentially participates in the wide variety of targets that are sensed by ALPS-containing proteins.

Beyond understanding membrane curvature sensing by AHs, the fluorescent probes we have designed and characterized are potentially useful to study dynamic cellular processes linked to specific organelle subdomains. In particular, the respective roles and dynamics of ER cisternae and ER tubules are not fully elucidated. Being able to specifically stain ER tubules may help resolving these issues. This could for instance allow assigning the location of specific proteins to specific ER domains. So far, this was achieved by 3D reconstruction of image stacks, or by thresholding methods applied to 3D stacks of confocal images [50]. Importantly, GFP^133^ did not show any obvious deleterious effect on cells, even at high expression levels: no growth or morphological defects were observed, and stable cell-lines could be established and maintained for several generations. As their soluble fraction is lower, the reinforced mutants (GFP^133^/V259F or GFP^133^/L265W) may be better ER tubules markers. As the ER is extremely sensitive to fixation and highly dynamic, the ability to follow its 3D structure in a 2D imaging set-up will likely improve the comprehension of its structure and function.

## Materials and Methods

### Plasmids

All GFP-tagged constructs were made in the pEGFP-N1 or pEGFP-C1 vectors (Clontech). The N-terminal fragment (AA 1–96) of human Sec61ß was cloned by PCR amplification using primers 5’-CTGGA**AGATCT**ATGCCTGGTCCGACCC-3’ and 5’-CTGGAGGTACCAGCGAACGAGTGTACTTGCCC-3’. The PCR products were then digested by BglII and KpnI (Takara), and ligated into pmCherry-N1 (Clontech) using the T4-DNA Ligase (Roche). The ACC-GFP construct was already described in [30]. Cloning of the AH of Sar1 or the ALPS motifs of Nup133 and GMAP210 in the expression vectors was done by insertion of the sequences into the appropriate vectors by mutagenesis (Quickchange Lightning Site-directed mutagenesis kit, Agilent technologies) following the protocol described by Geiser and collaborators [51]. S1 Table summarizes the details of all constructs made in this study. All point mutants were obtained using a site-directed mutagenesis kit (Quickchange Lightning Site-directed mutagenesis kit, Agilent technologies). All constructs were verified by DNA sequencing.

### Cell culture and transfection

U2OS cells were purchased from the ATCC (HTB-96). Cells were grown in DMEM (Gibco), supplemented with 10% FCS (BioWest) and an antibiotic, antimycotic mix (Zell Shield, Minerva biolabs). For live cell imaging, cells were grown in 8-well, cell culture-treated chambers (IBIDI). Otherwise, cells were plated on glass coverslips and incubated in 6-well plates. The day after seeding, cells were transfected using Lipofectamine 2000 reagent (Invitrogen) according to manufacturer’s recommendations. The medium was replaced with fresh medium 2–4 hours after transfection, and cells were imaged live or fixed 16-20h after transfection.

### Antibodies and reagents

The anti-GM130 (BD Transduction Laboratories) was used at a 1:250–1:400 dilution for immunofluorescence. Hoechst (Invitrogen) was used at 1μg/ml. The mitochondrial marker MitoTracker Red CMX (Invitrogen) was used at 100nM. The secondary donkey anti-Mouse coupled to Alexa594 (Invitrogen) was used at a 1:400 dilution.

### Immuno-fluorescence and imaging

As the ER structure is extremely sensitive to fixation, cells were imaged live when possible. For immuno-fluorescence, cells were fixed in 3% PFA, 0.5% glutaraldehyde for 20 minutes at room temperature. Cells were then saturated and permeabilized in IF buffer (10 mg/mL BSA, 0.1% Triton, 0.02% SDS in PBS) for 20 minutes at RT, incubated with an appropriate dilution of primary antibody in IF buffer for 1h at RT, rinsed 3 times in IF buffer, incubated for 1h at RT in secondary antibody diluted in IF buffer, rinsed three times in IF buffer, and an additional rinse in water. Coverslips were then mounted with Moewiol (Biovalley) onto glass slides.

All images were acquired on a Leica SP5 confocal microscope. For live cell imaging, the culture medium was supplemented with 2.5 mM HEPES pH 7.5 and cells were placed in a thermostatic chamber set at 37°C.

Image acquisition was performed on the imaging platform of IPMC CNRS and on workstations of the Montpellier RIO Imaging facility.

Images were analyzed with the FIJI software (http://fiji.sc/Fiji). Intensity profiles were obtained with the “Plot profile” tool, or a macro developed by F. Brau for multi-channel analysis.

For the t-test analysis, intensity profiles were obtained as described above. GFP and mCherry intensities of 10 consecutive pixels spanning cytoplasm, tubules or cisternae were compared using a paired Student t-test. The data were plotted using Prism.

The texture parameter “Contrast” as defined by Haralick [39] was obtained with an ImageJ GLCM Texture plug-in modified from Julio E. Cabrera’s plug-in. The texture parameters were computed for a 10 μm^2^ area in the peripheral ER region. All images were acquired with identical settings, in non-saturating conditions. Contrast is defined by Eq (1):

$$cont=\sum_{i=1}^{N}\sum_{j=1}^{N}{\left(i-j\right)}^{2}P(i,j)$$

where N is the number of grey levels, P(i,j) is the probability that a pixel of intensity i neighbours a pixel of intensity j.

As expression level of the fluorescent proteins may vary from cell to cell, we plotted the normalized contrast defined by Eq (2):

$$C=\frac{cont}{\mu^{2}}$$

where μ is the mean intensity of the analyzed area.

Contrast data were plotted with the Prism software.

## Supporting Information

S1 Fig (A) On the left is shown the crystal structure of the N-terminal domain of Nup133 (1XKS). Most of the ALPS motif is not resolved in the structure but is schematically represented by the dotted orange line. On the right is shown the crystal structure of EGFP (2Y0G), in which the ALPS motif has been schematized. The two crystal structures are shown at similar scales. (B) Schematic representation, roughly on scale, of GMAP210 and GMAP-ACC-GFP. Note that the long coiled-coil domain in GMAP210 is discontinuous and may harbour kinks. (C) Left panel: Crystal structure of ySar1 (2FA9). The N-terminal AH was truncated for expression and crystallisation, its position is schematized by the blue arrow. Right panel: EGFP structure in which Sar1 AH has been schematically added. Note that the two crystal structures are shown at similar scales. (D) Schematic representation of the nuclear pore membrane, ER tubule membrane and COP vesicle membrane. The three types of structures have similar positive curvature radii. Their total curvature can easily be sorted according to the curvature in the orthogonal direction (negative for pores, null for tubules and positive for vesicles).
(TIF)

S2 FigBriefly, Haralick’s texture parameters are based on the Grey Level Co-occurrence Matrix (GLCM): (i,j) coordinates of this matrix are the probability P(i,j) that a pixel of intensity i has a direct neighbour (in a given direction) of intensity j.Texture measures can be calculated from this matrix. For instance, the contrast (CON) is the sum of square variances: $CON=\sum_{i,j}{\left(i-j\right)}^{2}P(i,j)$ (1). For our study, we calculated normalized contrast values in peripheral areas of cells expressing different versions of the GFP^133^ protein, as defined by: $normCON=\frac{CON}{\mu^{2}}$ (2) where *μ* is the mean intensity of pixels in the region of interest. This eliminates the bias introduced in contrast comparison when images have dissimilar pixel intensities. A proof of principle of this method is illustrated in S2 Fig. (A) Synthetic images modelling a soluble (left) or ER specific (right) fluorescent protein. The middle panel mimics a protein with soluble and ER fractions. (B) Contrast values calculated from the Haralick’s GLCM matrix extracted from the synthetic images in (A) (left). In the right panel, contrast values were normalized to the squared mean fluorescence level of the image. As all images were generated with a similar total fluorescence level, normalization does not affect the analysis. (C) Confocal images of cells expressing GFP (left) or the ER-specific Sar1-GFP (right). Haralick’s texture was analyzed in a 10μm^2^ area in the cell periphery. Zoomed views of the framed areas showing the pixel details are shown below. (D) Haralick’s contrast values of the framed areas in the left panels were calculated. In cells of similar expression levels, an ER-specific protein has a lower level at the periphery, due to the high concentration of ER in the nuclear vicinity. In contrast, a soluble protein has a homogenous level all over the cell. As contrast values are weighted by squared pixel intensities, contrast of a small portion of a GFP-expressing cell is higher than for a Sar1-GFP cell. To alleviate this bias, contrast values were normalized to the squared mean intensities of the given areas.(TIF)

S3 Fig (A) Intensity plots of GFP133 / I265F & Sec61-mCherry along a line spanning an ER tubule and an ER flat sheet. (B) Detail of a U2OS cell co-expressing GFP133 / I265F and Sec61-mCherry. A portion of the nuclear envelope surface (upper left) and flat cisternae are visible. (C) Intensity plots of GFP133 / V259F & Sec61-mCherry along a line spanning an ER tubule and an ER flat sheet. (D) Detail of a U2OS cell co-expressing GFP133 / V259F and Sec61-mCherry. A portion of the nuclear envelope surface (upper right) and flat cisternae are visible. (E) Volume of hydrophobic residue side chains (from [40]). These values were used to estimate volume ratios in Fig 4E. (F-H) Western Blot of total extracts from cells transfected by GFP^133^ or mutants. Scale bars are 10μm.
(TIF)

S4 Fig (A) U2OS cells were transfected with GFP^133^, 133-GFP or 133-ACC-GFP. Cells were treated with MitoTracker for 15 minutes. Medium was then replaced with fresh medium, and cells were imaged live with a confocal microscope. (B) Confocal images of live cells co-transfected with Sec61-mCherry (red) and GFP-GMAP-GMAP (green). Scale bars are 10μm. (TIF)

S5 Fig (A) Confocal images of live cells transfected with GMAP-ACC-GFP or ACC-GFP. Top panels show single Z-slices; bottom panels are maximum projections of Z-stacks spanning the entire cell (1 stack / 300nm). (B) Confocal images of cells transfected with GMAP-ACC-GFP or GMAP-shortACC-GFP, fixed and stained with anti-GM130 (red). Scale bars are 10μm.
(TIF)

S6 Fig Schematic to illustrate how the presence of the coiled-coil domain can prevent binding of an AH to a flat membrane: the left panel shows how, when monomeric, Sar1-GFP can bind to flat membranes. In the middle panel, the formation of a putative angle following dimerization of Sar1-ACC-GFP is unfavourable for flat membrane binding. In contrast, this type of structure adapts well with a curved structure (vesicle or tubule), as illustrated in the right panel.
(TIF)

S1 Table Details of the different constructs used in the study. The amino-acid sequences of the peptides fused to EGFP are listed; their position relative to EGFP is indicated in the “Position” column. Sequences of the linkers are in italic, when it applies.
(DOCX)

## Abbreviations List

AH: Amphipathic Helix
ALPS: Amphipathic Lipid Packing Sensor
BAR: Bin-Amphiphysin-Rvs
EGFP: Enhanced Green Fluorescent Protein
ER: Endoplasmic Reticulum
NE: Nuclear Envelope
NPC: Nuclear Pore Complex
SIM: Structured Illumination Microscopy
