# Supplementary figures and images for: Membrane Curvature Sensing by Amphipathic Helices Is Modulated by the Surrounding Protein Backbone
Source: PLoS One. 2015 Sep 14;10(9):e0137965. doi: 10.1371/journal.pone.0137965 (PMC4569407; doi:10.1371/journal.pone.0137965)

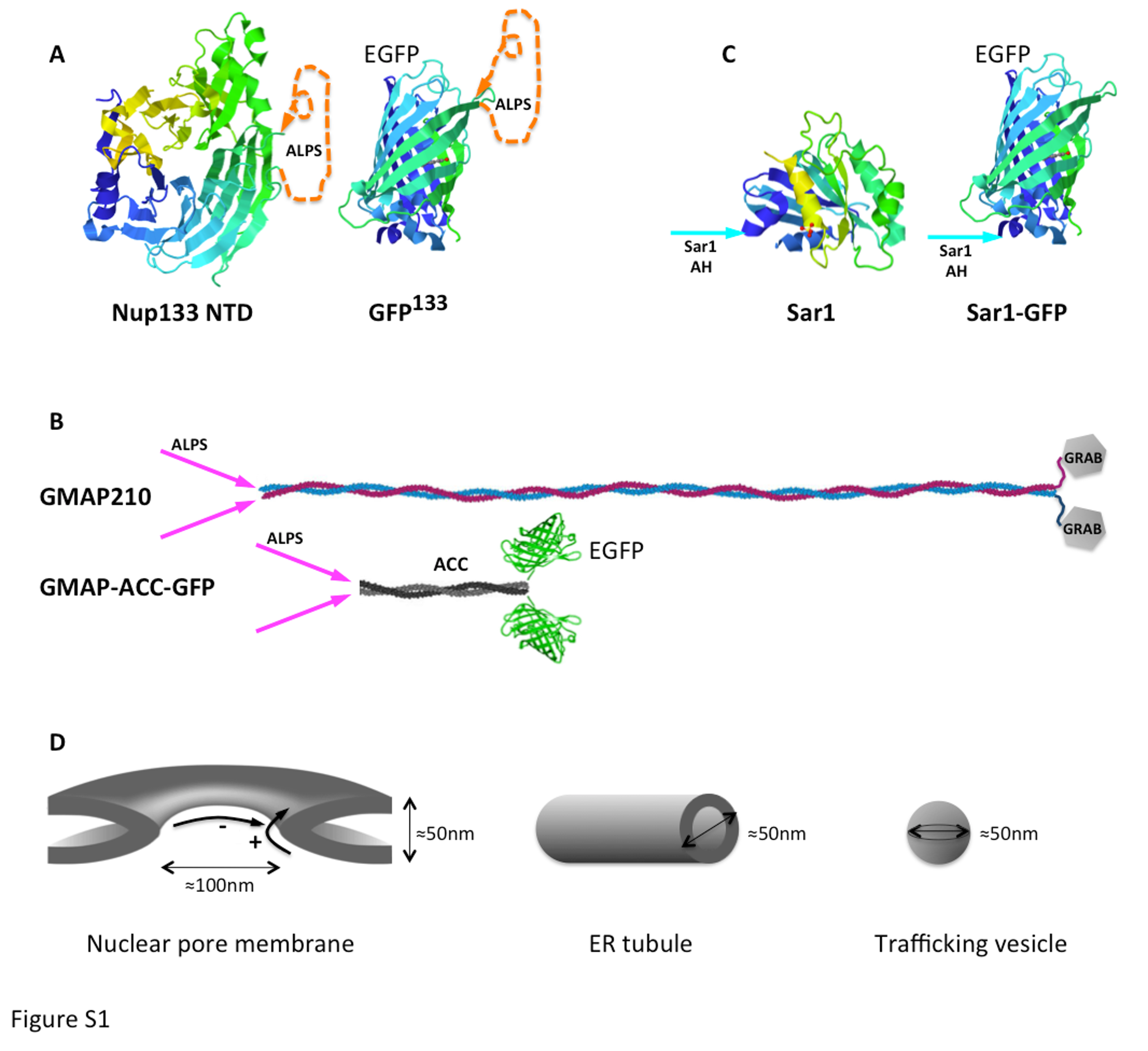

Supplement: S1 Fig — The three types of structures have similar positive curvature radii. Their total curvature can easily be sorted according to the curvature in the orthogonal direction (negative for pores, null for tubules and positive for vesicles). (TIF) [file pone.0137965.s001.tif]

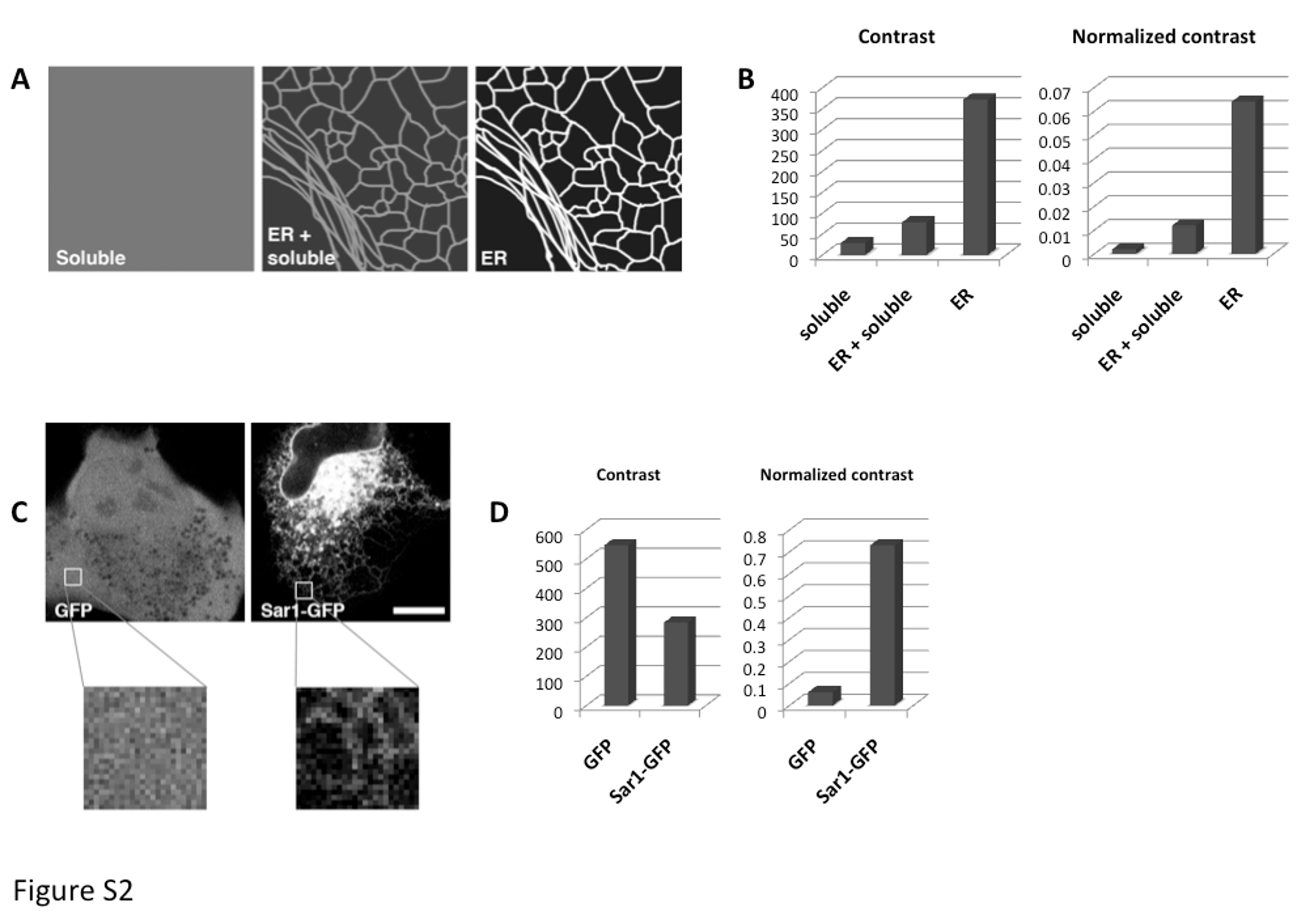

Supplement: S2 Fig — Texture measures can be calculated from this matrix. For instance, the contrast (CON) is the sum of square variances: CON=∑i,j(i−j)2P(i,j) (1). For our study, we calculated normalized contrast values in peripheral areas of cells expressing different versions of the GFP133 protein, as defined by: normCON=CONμ2 (2) where μ is the mean intensity of pixels in the region of interest. This eliminates the bias introduced in contrast comparison when images have dissimilar pixel intensities. A proof of principle of this method is illustrated in S2 Fig. (A) Synthetic images modelling a soluble (left) or ER specific (right) fluorescent protein. The middle panel mimics a protein with soluble and ER fractions. (B) Contrast values calculated from the Haralick’s GLCM matrix extracted from the synthetic images in (A) (left). In the right panel, contrast values were normalized to the squared mean fluorescence level of the image. As all images were generated with a similar total fluorescence level, normalization does not affect the analysis. (C) Confocal images of cells expressing GFP (left) or the ER-specific Sar1-GFP (right). Haralick’s texture was analyzed in a 10μm2 area in the cell periphery. Zoomed views of the framed areas showing the pixel details are shown below. (D) Haralick’s contrast values of the framed areas in the left panels were calculated. In cells of similar expression levels, an ER-specific protein has a lower level at the periphery, due to the high concentration of ER in the nuclear vicinity. In contrast, a soluble protein has a homogenous level all over the cell. As contrast values are weighted by squared pixel intensities, contrast of a small portion of a GFP-expressing cell is higher than for a Sar1-GFP cell. To alleviate this bias, contrast values were normalized to the squared mean intensities of the given areas. (TIF) [file pone.0137965.s002.tif]

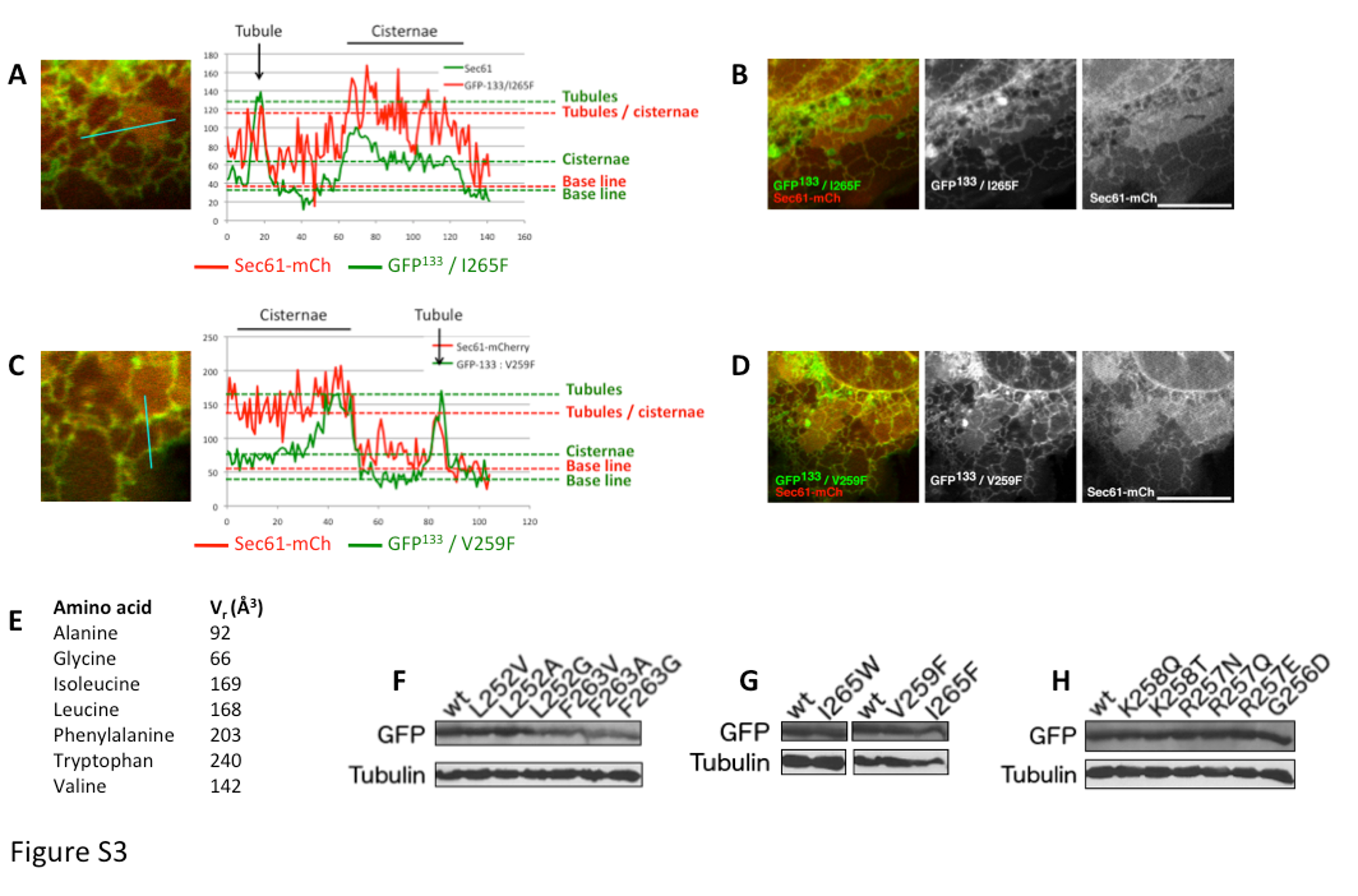

Supplement: S3 Fig — These values were used to estimate volume ratios in Fig 4E. (F-H) Western Blot of total extracts from cells transfected by GFP133 or mutants. Scale bars are 10μm. (TIF) [file pone.0137965.s003.tif]

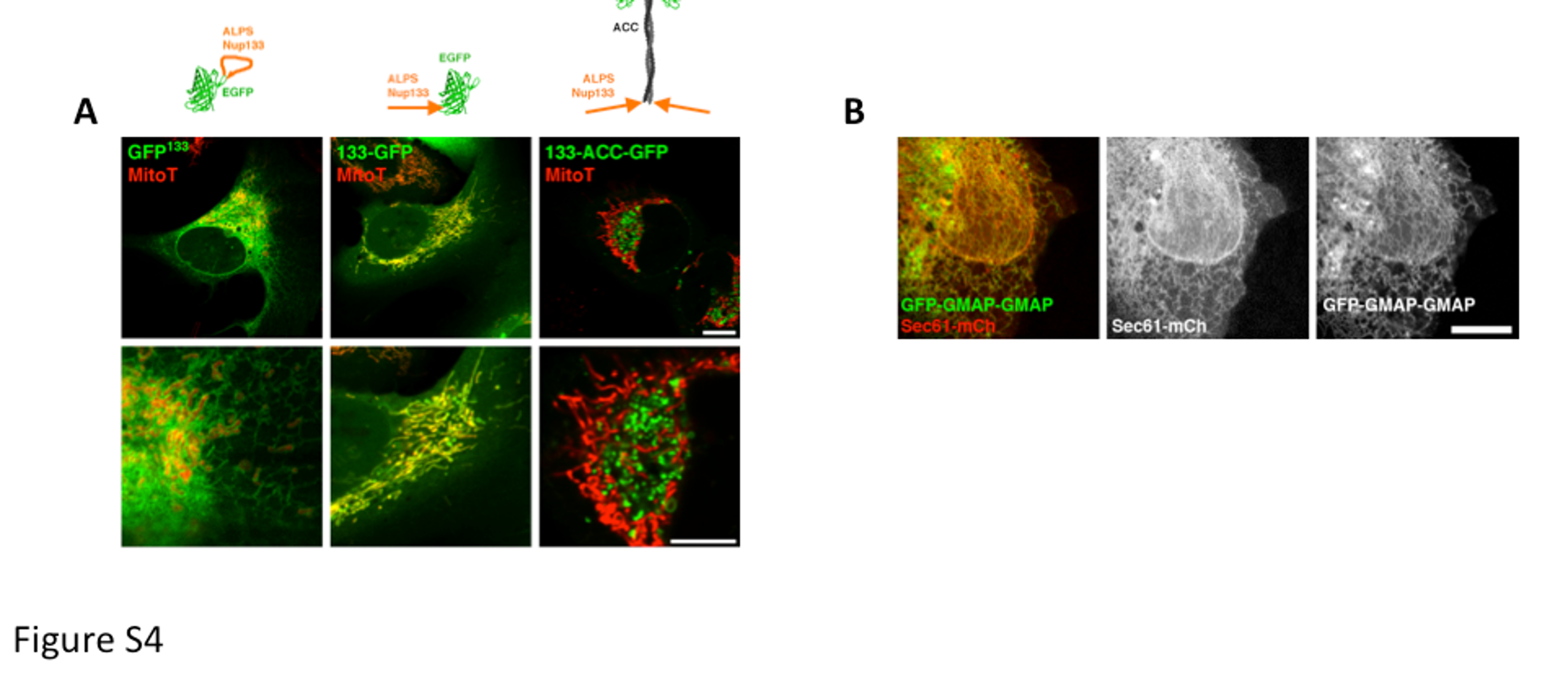

Supplement: S4 Fig — (TIF) [file pone.0137965.s004.tif]

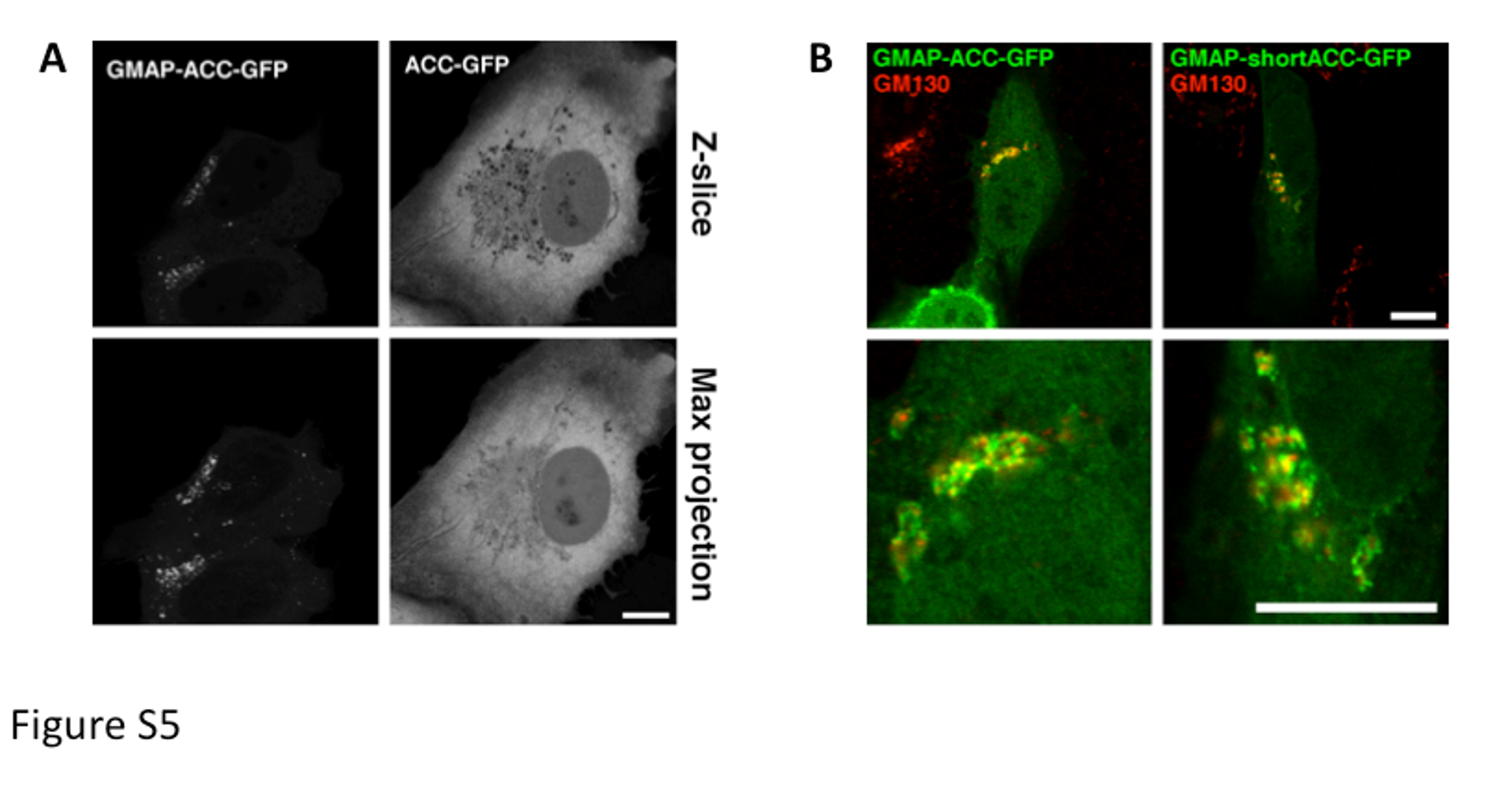

Supplement: S5 Fig — Scale bars are 10μm. (TIF) [file pone.0137965.s005.tif]

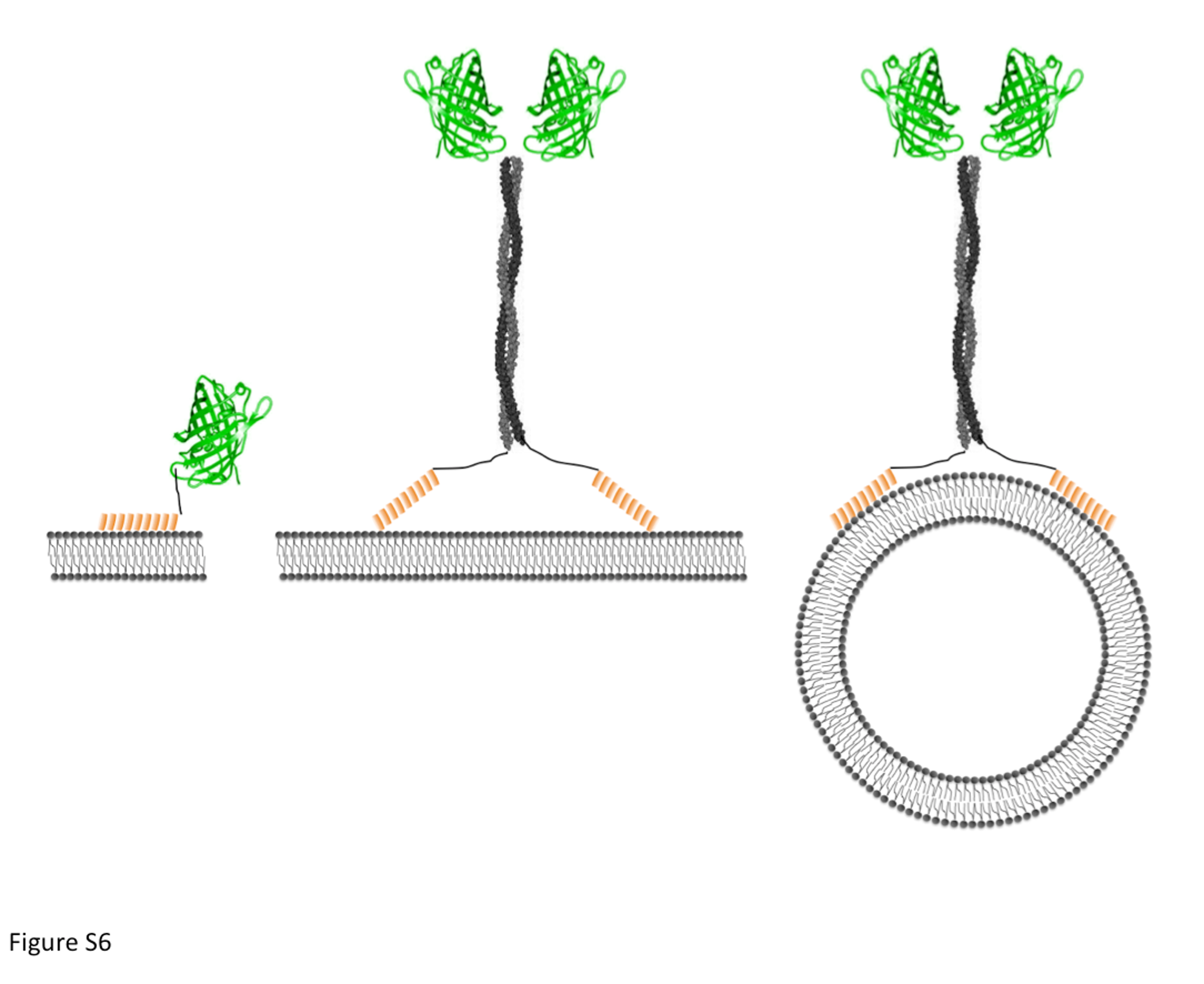

Supplement: S6 Fig — In the middle panel, the formation of a putative angle following dimerization of Sar1-ACC-GFP is unfavourable for flat membrane binding. In contrast, this type of structure adapts well with a curved structure (vesicle or tubule), as illustrated in the right panel. (TIF) [file pone.0137965.s006.tif]
