# Supplementary material for: Membrane Curvature Sensing by Amphipathic Helices Is Modulated by the Surrounding Protein Backbone
Source: PLoS One. 2015 Sep 14;10(9):e0137965. doi: 10.1371/journal.pone.0137965 (PMC4569407; doi:10.1371/journal.pone.0137965)
Supplement: S1 Table — The amino-acid sequences of the peptides fused to EGFP are listed; their position relative to EGFP is indicated in the “Position” column. Sequences of the linkers are in italic, when it applies. (DOCX) [file pone.0137965.s007.docx]

| **Construct name** | **Insert sequence** | **Position** | **Vector used** |
| --- | --- | --- | --- |
| **GMAP-ACC-GFP** | MSSWLGGLGSGLGQSLGQVGGSLASLTGQISNFTKDML-*GSAT-* | N-terminus of ACC-EGFP | ACC-EGFP |
| **GFP^133^** | -LPQGQGMLSGIGRKVSSLFGILS- | Between AA I172 and G175 of EGFP | pEGFP-N1 |
| **Sar1-GFP** | MAGWDIFGWFRDVLASLGLWNKH-*PVAT-* | N-terminus of EGFP | pEGFP-N1 |
| **133-GFP** | MQGQGMLSGIGRKVSSLFGILSPS-*PVAT-* | N-terminus of EGFP | pEGFP-N1 |
| **GFP-133** | *-SGLRSRAQASNS*-LPQGQGMLSGIGRKVSSLFGILS* | C-terminus of EGFP | pEGFP-C1 |
| **GFP-GMAP** | *-SGLRSRAQASNS-*MSSWLGGLGSGLGQSLGQVGGSLASLTGQISNFTKDML* | C-terminus of EGFP | pEGFP-C1 |
| **GFP-GMAP-GMAP** | *-SGLRS*-MSSWLGGLGSGLGQSLGQVGGSLASLTGQISNFTKDML-*SGLGNS*-MSSWLGGLGSGLGQSLGQVGGSLASLTGQISNFTKDML* | C-terminus of EGFP | pEGFP-C1 |
| **Sar1-ACC-GFP** | MAGWDIFGWFRDVLASLGLWNKH-*GSAT-* | N-terminus of ACC-EGFP | ACC-EGFP |
| **133-ACC-GFP** | MLPQGQGMLSGIGRKVSSLFGILS-*GSAT-* | N-terminus of ACC-EGFP | ACC-EGFP |
|  |  |  |  |
